# Neutralizing Antibody against Enterovirus D68 in Children and Adults before 2014 Outbreak, Kansas City, Missouri, USA^1^

**DOI:** 10.3201/eid2503.180960

**Published:** 2019-03

**Authors:** Christopher J. Harrison, William C. Weldon, Barbara A. Pahud, Mary Anne Jackson, M. Steven Oberste, Rangaraj Selvarangan

**Affiliations:** The Children’s Mercy Hospital, Kansas City, Missouri, USA (C.J. Harrison, B.A. Pahud, M.A. Jackson, R. Selvarangan);; University of Missouri at Kansas City, Kansas City (C.J. Harrison, B.A. Pahud, M.A. Jackson, R. Selvarangan);; Centers for Disease Control and Prevention, Atlanta, Georgia, USA (W.C. Weldon, M.S. Oberste)

**Keywords:** enterovirus, EV-D68, neutralizing antibody, children, adults, age, seroprevalence, Fermon, Kansas City, Missouri, United States, outbreak, 14-18949, 14-18952, 14-18953, viruses, respiratory infections, race/ethnicity, Hispanic, microneutralization, viruses

## Abstract

We evaluated enterovirus D68 seroprevalence in Kansas City, Missouri, USA, from samples obtained during 2012–2013. Neutralizing antibodies against Fermon and the dominant 2014 Missouri isolate were universally detected. Titers increased with age. Widespread circulation of enterovirus D68 occurred before the 2014 outbreak. Research is needed to determine a surrogate of protection.

The first enterovirus D68 (EV-D68) isolate (Fermon) was identified in 1962 (*1*,*2*). Before a 2014 EV-D68 outbreak, US reports of EV-D68 were relatively sparse (<100 sporadic cases and periodic outbreaks in 50 years) (*3*). In autumn 2014, a total of 1,153 confirmed EV-D68 cases occurred in 49 states and the District of Columbia; EV-D68 patients mostly had respiratory symptoms consistent with those in previous EV-D68 outbreaks and cases (*4*). Nationwide, severe disease occurred mostly in school-aged children. Through September 2014, EV-D68 was detected in 338 of 551 children with rhinovirus/enterovirus-positive test results; most (61.3%) were hospitalized at The Children’s Mercy Hospital (Kansas City, MO, USA). Hospitalized EV-D68 patients often had asthma or recurrent wheezing. Many of these EV-D68–infected children had unusually severe, refractory bronchospasms, which resulted in 100 intensive care unit stays (*5*).

## The Study

We assessed the prevalence of EV-D68 neutralizing antibody in the Kansas City population before the 2014 outbreak using deidentified banked serum samples (stored at The Children’s Mercy Hospital) collected in 2012 (n = 155) and 2013 (n = 281) from healthy persons >2 years of age during a poliovirus seroprevalence study (*6*). Age, sex, and race/ethnicity distributions matched 2010 Kansas City census data (Appendix) (*6*).

**Figure 1:**
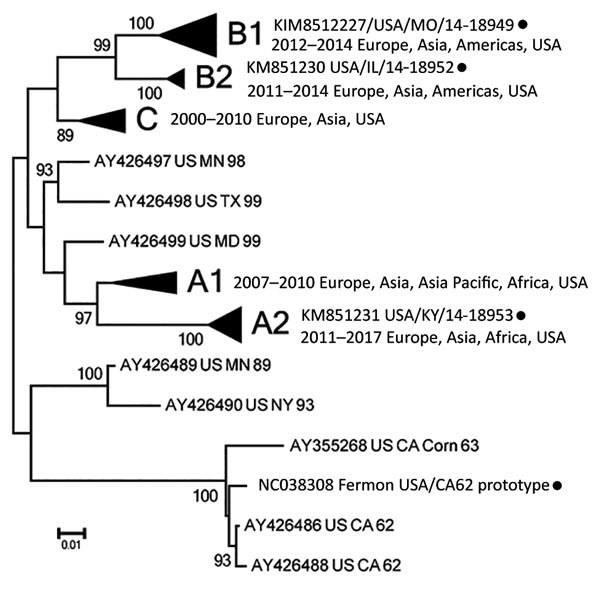
Unrooted neighbor-joining tree depicting phylogenetic relatedness of reference enterovirus D68 isolates (black circles) used in microneutralization assays in study of enterovirus D68 seroprevalence among children and adults in Kansas City, Missouri, USA, 2012–2013. We constructed tree using complete virus protein 1 gene sequences and MEGA6.0 (*8*). Branching within major clades (bolded) is collapsed for clarity.

We performed serology testing at the Centers for Disease Control and Prevention (Atlanta, Georgia, USA). We used an adapted poliovirus microneutralization assay to test samples for neutralizing antibodies (*6*,*7*) against 4 phylogenetically distinct EV-D68 isolates: Fermon (GenBank accession no. NC038308); the 2014 Missouri isolate 14-18949 (clade B1, GenBank accession no. KM851227); and 2 related but non-Missouri 2014 isolates, 14-18952 (clade B2) and 14-18953 (clade A2; GenBank accession nos. KM851230–1; Figure 1; Appendix) (*9*). The proportion of US patients from whom these three 2014 circulating strains were detected was >91% for 14-18949, 7.4% for 14-18952, and <2% for 14-18953 (*10*).

Besides age, sex, and race/ethnicity (Hispanic vs. non-Hispanic), population demographics are descriptive; small subset numbers precluded formal statistical analysis. We performed analyses using Sigmaplot version 12.2 (http://www.sigmaplot.co.uk/index.php; Appendix), and we considered p values <0.05 significant.

Of 436 serum samples, 217 were from male donors; median age was 13 (range 2–81) years. All had neutralizing antibody (i.e., >3 log_2_, >1:8 titer) against Fermon and 14-18949 (Table); 97% of samples had neutralizing antibody to 14-18953 and 89% to 14-18952. Overall seropositivity for the 4 isolates was not different (p = 0.763).

**Table:** Neutralizing antibody positivity and titers for each enterovirus D68 isolate, by age group, Kansas City, Missouri, 2012–2013

Age group, y	No.	% Neutralizing antibody positive; median (range) neutralizing antibody titer*			
		Fermon	14-18949	14-18952	14-18953
2–5	79	100; 5.50 (3.17–9.5)	100; 5.83 (3.5–10.5)	60; 3.17 (2.5–10.5)	81; 4.17 (2.5–10.5)
6–10	97	100; 6.17 (3.17–10.5)	100; 7.83 (4.17–10.5)	89; 7.83 (2.5–10.5)	83; 6.17 (2.5–10.5)
11–15	91	100; 5.83 (3.17–10.5)	100; 7.83 (3.17–10.5)	97; 8.50 (2.5–10.5)	93; 6.50 (2.5–10.5)
16–50	84	100; 8.50 (3.83–10.5)	100; 8.50 (4.83–10.5)	98; 9.17 (2.5–10.5)	96; 7.17 (2.5–10.5)
>50	85	100; 10.50 (6.5–10.5)	100; 8.83 (4.83–10.5)	99; 9.50 (2.5–10.5)	98; 6.83 (2.5–10.5)
Total	436	100; 6.83 (2.83–10.5)	100; 7.83 (3.5–10.5)	89; 8.34 (2.5–10.5)	91; 6.50 (2.5–10.5)

*Antibody titers were measured by using the cell viability kit ATPlite (Perkin Elmer, http://www.perkinelmer.com); the titers shown are the log_2_ inverse dilution of the lowest antibody concentration with luminescent activity.

In total, 50% (24/48) of the 14-18952–seronegative samples and 57% (24/42) of the 14-18953–seronegative samples (p<0.001) came from male donors. Our 2–5-year-old age group made up the largest proportions of these seronegative populations (67% [32/48] of 14-18952 and 36% [15/42] of 14-18953; p = 0.003). The 21 persons seronegative for both 14–18952 and 14–18953 had a median age of 3 (2–61) years. Donor sex and race/ethnicity and season of sample acquisition did not differ between samples seropositive and seronegative for 14-18952 or 14-18953 (data not shown).

Neutralizing antibody titers also did not differ by season of sample acquisition, donor race/ethnicity, or donor sex. Mean titers to 14-18949 were lower (p<0.05) among self-identifying Hispanics (n = 36; 7.1 log_2_, range 3.83–10.5 log_2_) than among non-Hispanics (n = 400; 7.83 log_2_, range 3.17–10.5 log_2_), but this difference might not be clinically significant. Median titers rose with each advancing age group, except against Fermon among 11–15-year-olds (p<0.001; Table). The overall median titer was highest for 14-18952 (8.34 log_2_, range 2.5–10.5 log_2_; p<0.001), despite a comparatively lower seropositivity (89%). The overall titer was lowest for 14-18953 (6.83 log_2_, range 2.83–10.5 log_2_; p<0.001), despite a relatively high seropositivity (91%).

**Figure 2:**
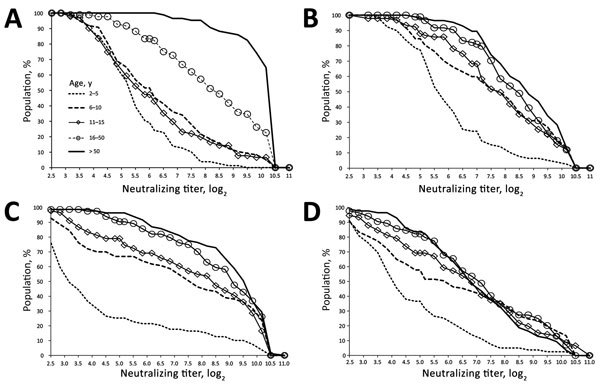
Reverse cumulative distribution curves of neutralizing antibody titers against enterovirus D68 isolates, by isolate and age group, Kansas City, Missouri, USA, 2012–2013. A) Fermon; B) dominant 2014 isolate 14-18949; C) less frequently circulating 2014 isolate 14-18952; D) rare 2014 isolate 14-18953. The reverse cumulative distribution pattern for 14-18949 varied the least by age group.

All serum samples had neutralizing antibody against the major EV-D68 isolates circulating in the United States in 2014, and most had antibody to the other 2 less frequently detected isolates that year (Figure 2; Appendix Figure). During the 2014 outbreak, 5–10-year-olds (who would have been 3–8-year-olds during the sampling time of our study) had the most severe disease. Severe EV-D68 disease occurred often in children with atopic disease, reactive airway disease, or asthma. In 2014, little EV-D68 disease was noted among adolescents, adults, or the elderly (*4*). The age-associated severe EV-D68 respiratory disease observed in 2014 parallels our finding of lower overall titers in 2–5-year-olds and 6–10-year-olds.

Although introduction of EV-D68 into naive populations could have explained the 2014 outbreak, universal detection of antibody against 14-18949 (dominant 2014 isolate) before 2014 indicates previous widespread exposure to 14-18949 or a related isolate. EV-D68 was also detected in Kansas City as early as 2009 (F. Hassan, University of Missouri at Kansas City, pers. comm., May 2018). Thus, the Kansas City 2014 outbreak did not occur because of population naivete to a 14-18949–like isolate. Indeed, neutralizing antibodies to other EV-D68 isolates were also detected (*4*,*10*). That 2–5-year-olds in our study had lower titers to 14-18949 (Figure 2) suggests that older persons had more experience with 14-18949 or confirms that antibody elicited by non–14-18949 isolates can also neutralize 14-18949. Severe disease during the 2014 outbreak occurred among children who, according to our results, were relatively experienced with this pathogen; they were positive for neutralizing antibodies against 14-18949 but had lower median titers and a reduced reverse cumulative distribution compared with other age groups. 

Why this large outbreak was able to occur in a population with a high prevalence of neutralizing antibody against the outbreak isolates remains unclear. One possibility is that respiratory tract mucosal antibody (probably in the form of secretory IgA) is more relevant than serum antibody for protection against respiratory disease (*11*). In this study, we could not address this possibility because only serum samples were available for testing. Also, certain persons could be more susceptible to severe disease because of genetic factors, preexisting atopy or asthma, or differences in other parts of the immune response, including immunopathologic responses. The argument for multiple factors contributing to disease despite the presence of neutralizing antibody is bolstered by the predilection of persons with asthma or atopic disease to have severe disease (*5*); further, asthma patients experience more tight junction injury than persons without asthma during rhinovirus infection (*12*).

The only demographic factor potentially affecting titers was Hispanic race/ethnicity. However, the ≈0.8 log_2_ difference might not be clinically significant, considering the median titers in both groups were >5 log_2_; thus, whether race/ethnicity is a factor is unclear.

Our data parallel another study with a similar 2011 sampling timeframe conducted in China (*13*). In that study, neutralizing titers against Beijing/2008/01 EV-D68 were low in serum samples collected in 2004 for all age groups, but their 2011 titers resembled our data, despite few reported EV-D68 illnesses in the sampled area during 2007–2011. Their lowest overall titers were also observed in persons of younger age groups.

EV-D68 has been proposed as a cause of acute flaccid myelitis. Increasing reports of this condition underscore the need to better understand EV-D68 seroprevalence and circulation (*14*).

Our study had several limitations (Appendix). Because of the retrospective study design, our data and interpretations are limited regionally and temporally. We tested for only 4 select EV-D68 isolates, and antibody reactivity with other isolates might differ.

## Conclusions

We detected at least some neutralizing antibody to Fermon and the dominant 2014 isolate (14-18949) in all 436 EV-D68 samples acquired during 2012–2013 in Kansas City. Prospective studies are warranted to define a protective threshold of serum neutralizing antibody (or a surrogate of protection), the distribution of titers in children <2 years of age, and whether antibody levels differ by race/ethnicity.

Appendix Additional information on neutralizing antibodies against enterovirus D68 in children and adults in Kansas City, Missouri, USA, 2012–2013.
