# Supplementary material for: Neutralizing Antibody against Enterovirus D68 in Children and Adults before 2014 Outbreak, Kansas City, Missouri, USA
Source: Emerg Infect Dis. 2019 Mar;25(3):585–8. doi: 10.3201/eid2503.180960 (PMC6390745; doi:10.3201/eid2503.180960)
Supplement: Appendix — Additional information on neutralizing antibodies against enterovirus D68 in children and adults in Kansas City, Missouri, USA, 2012–2013. [file 18-0960-Techapp-s1.pdf]

Article DOI: https://doi.org/10.3201/eid2503.180960

Neutralizing Antibody against Enterovirus D68 in Children and Adults before 2014 Outbreak, Kansas City, Missouri, USA

Appendix

Microneutralization Assay

The microneutralizion assay was adapted from an established poliovirus antibody assay (*1*,*2*). In brief, 2-fold serum dilutions, 1:8 to 1:1,024, were combined with 100 cell culture ID_{50} of enterovirus D68 (EV-D68) to enable antibody to bind to virus. After a 3-hour incubation, each virus–serum mixture was inoculated onto rhabdomyosarcoma (CCL-136; American Type Culture Collection, https://www.atcc.org) cell monolayers. Each serum dilution was tested in triplicate against each isolate. Each run had a known positive control serum (horse anti-Fermon antibody), and multiple (at least 4) positive control replicates were distributed across each run. When >7 serum samples were tested in the same run, sample position was randomized via a balanced block randomization scheme. Each run included 2 control plates with no serum or control antibody; rhabdomyosarcoma cells alone served as a no-virus control. A back-titration virus–control plate was used for each of the 4 EV-D68 isolates to confirm the amount of antigen used in each run. A luminescent cell viability kit (ATPlite; Perkin Elmer, http://www.perkinelmer.com) was used to evaluate neutralization. Samples with luminescent activity at a titer of greater than 3 $\log_2$ (1:8 dilution) were considered positive for neutralizing antibody.

Demographics and Data Analysis

We used the following age groups: 2–5-year-olds (n = 79), 6–10-year-olds (n = 97), 11–15-year-olds (n = 91), 16–50-year-olds (n = 84), and 51–81-year-olds (n = 85). Race/ethnicity was self-reported, and for analysis, we used the groups white (n = 330), black (n = 66), mixed race (n = 19), other (n = 10), Asian (n = 7), Native American (n = 5), and Pacific Islander (n =

1). In total, 36 participants were Hispanic/Latino and 396 were non-Hispanic/Latino; 10 declined to report race, and 6 declined to give ethnicity.

For analysis, the population was age stratified. The age and sex distribution and season of sample acquisition were not different between Hispanics and non-Hispanics. Categorical values were analyzed by the χ^2 test. Overall antibody titers were analyzed by the Kruskal-Wallis rank-sum test to determine if values significantly differed between groups, with subset comparisons performed by using the Kolmogorov-Smirnov test. Differences between isolates were assessed by using nonparametric analysis of variance, and adjustments for multiple comparisons was performed by using Tukey-Kramer comparisons.

Reverse Cumulative Distribution

For the Fermon isolate, the titers of the >50-year-old age group were significantly higher than those of the 4 younger age groups (p<0.001 for all; Figure 1, panel A, main text). Likewise, the titers of the 16–50-year-old age group were higher than those of the 3 younger age groups (p<0.001 for all 3). The titers to 14–18949 among >50-year-olds were similar to those of the 16–50-year-olds but higher than those of the 3 youngest age groups (p<0.001 for all; Figure 1, panel B, main text). The 14–18949 titers among 2–5-year-olds were significantly lower than those of all other age groups. Titers to 14–18952 (Figure 1, panel C, main text) and 14–18953 (Figure 1, panel D, main text) were significantly lower in the 2–5-year-old age group than the 4 older age groups (p<0.001 for all). Titers to 14–18952 were also lower in the 6–10-year-old age group than the 3 oldest age groups (p<0.001 for all 3).

Comparisons of isolate seropositivity by age group (Appendix Figure, panels A–E) revealed that 2–5-year-olds had higher titers for both Fermon and 14–18949 than 14–18952 and 14–18953 (p<0.001 for all; Appendix Figure, panel A). In the 6–10-year-old age group, titers for 14–18949 were higher than those for both Fermon and 14–18953 but not different from those for 14–18952 (Appendix Figure, panel B). In the 11–15-year-old age group, titers for 14–18949 and 14–18952 did not differ but were higher than those for both Fermon and 14–18953 (p<0.001 for all; Appendix Figure, panel C). In the 16–50-year-old age group, the only difference noted was that titers for 14–18953 were lower than those for the other 3 isolates (p<0.001 for all; Appendix Figure, panel D). In the >50-year-old age group, titers for Fermon were higher than those for the

other 3 isolates, and titers for 14–18953 were lower than those for the other 3 isolates ($p<0.001$ for all; Appendix Figure, panel E).

Limitations

Limitations of our study include the retrospective study design and the use of deidentified samples stored since 2012–2013. We were not able to determine if seroprevalence differed at an earlier time point, e.g., in 2004 as in the study conducted in China (*3*). We have no clinical data for serum sample donors, e.g., information regarding comorbidities, such as asthma and atopy, or whether they got EV-D68 illness during 2014. We tested for neutralizing antibody against only 4 EV-D68 isolates, so patterns of neutralizing activity against other EV-D68 isolates could differ. However, we did test for the isolates known to have circulated in Kansas City, Missouri, USA, in 2014. Age ranges for our groups could be considered arbitrary; the age groups we used were adapted from those originally chosen to evaluate seroprevalence of poliovirus, another enterovirus. Our age groups parallel those in the Beijing report (*3*). We had no samples from children <2 years of age, who would most likely have been EV-D68 naive. The racial, ethnic, and age distributions of our population matched those of Kansas City census data and, therefore, might not be generalizable to other geographic areas. That said, these distributions closely mirrored those of the United States as a whole in 2010.

References

1. Wallace GS, Pahud BA, Weldon WC, Curns AT, Oberste MS, Harrison CJ. Seroprevalence of poliovirus antibodies in the Kansas City metropolitan area, 2012–2013. Hum Vaccin Immunother. 2017;13:776–83. PubMed http://dx.doi.org/10.1080/21645515.2016.1255386

2. Weldon WC, Oberste MS, Pallansch MA. Standardized methods for detection of poliovirus antibodies. In: Martin JM, editor. Methods in molecular biology. Vol. 1378. New York: Humana Press; 2016. p. 145–76.

3. Xiang Z, Li L, Ren L, Guo L, Xie Z, Liu C, et al. Seroepidemiology of enterovirus D68 infection in China. Emerg Microbes Infect. 2017;6:e32. PubMed http://dx.doi.org/10.1038/emi.2017.14

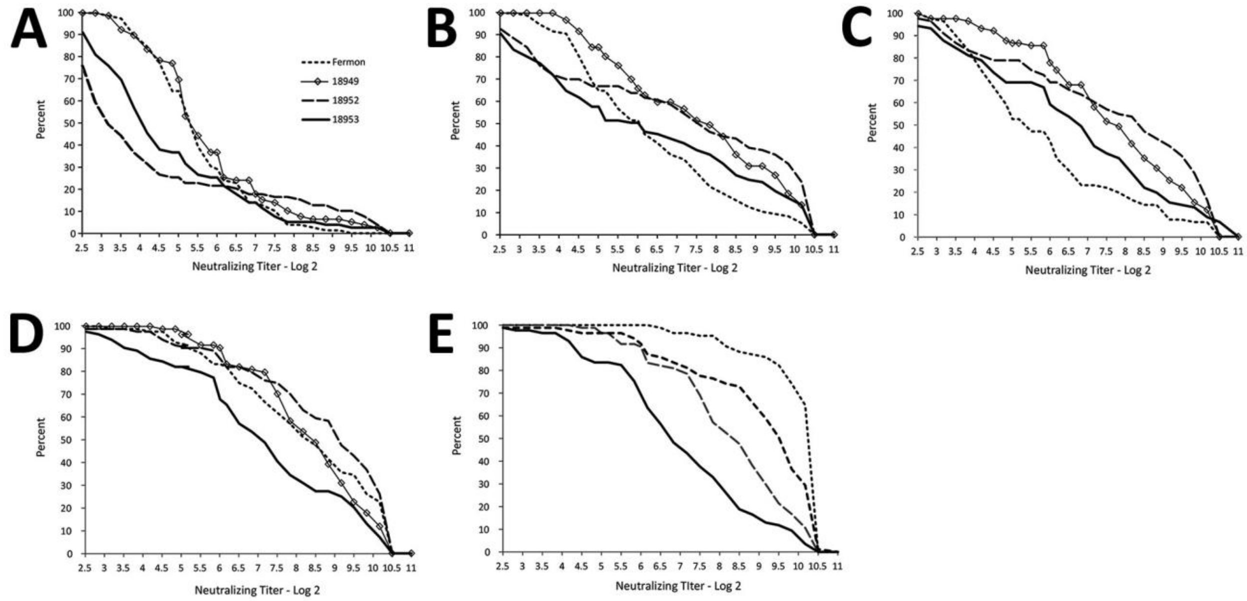

Appendix Figure. Reverse cumulative distribution (RCD) curves of enterovirus D68 (EV-D68) neutralizing antibody titers against 4 EV-D68 isolates, by age group, Kansas City, Missouri, USA, 2012–2013. A titer >3.0 $\log_2$ was considered positive for neutralizing antibody. RCDs represent the proportion of the population with a titer at least as high as the value on the *x* axis. Data from A) 2–5-year-olds; B) 6–10-year-olds; C) 11–15-year-olds; D) 15–50-year-olds; and E) >50-year-olds. Persons in the middle 3 age groups (6–50-year-olds) had the most similar RCD patterns.
